# 3,3'-Diindolylmethane plus Eflornithine suppress DNA Replication and Cell Cycle in Esophageal Squamous Cell Carcinoma *in vivo*

**DOI:** 10.7150/jca.65506

**Published:** 2022-05-16

**Authors:** Fayang Ma, Fangfang Liu, Wenna Nie, Kyle Laster, Xueli Tian, Bingbing Lu, Zushi Geng, Ruihua Bai, Dong Joon Kim, Kangdong Liu, Zigang Dong

**Affiliations:** 1 Department of Pathophysiology, School of Basic Medical Sciences, College of Medicine, Zhengzhou University, Zhengzhou, Henan, 450008, China.; 2 China-US (Henan) Hormel Cancer Institute, Zhengzhou, Henan, China.; 3 Department of Thyroid Surgery, The First Affiliated Hospital of Zhengzhou University, Zhengzhou, Henan, China.; 4 The Affiliated Cancer Hospital of Zhengzhou University, Zhengzhou, Henan, China.

**Keywords:** Esophageal squamous cell carcinoma, 3, 3'-diindolylmethane, eflornithine, proteomics

## Abstract

Esophageal squamous cell carcinoma (ESCC) is a malignant cancer that is responsible for a high mortality rate; it accounts for approximately 90% of the 456,000 esophageal cancer (EC) cases diagnosed annually. Effective natural or synthesized compounds to prevent, treat, and/or inhibit ESCC relapse are desperately needed. The natural di-indole compound 3,3'-diindolylmethane (DIM) is abundant in cruciferous vegetables and shows potent anti-tumor effects in multiple cancers. The synthesized Eflornithine (DFMO) is clinically used to treat African sleeping sickness. We demonstrated that the combination of DIM+DFMO could significantly suppress the ESCC growth in the* in vivo* study of three patient-derived xenograft (PDX) cases. Then, the corresponding underlying anticancer mechanisms were investigated via the isobaric tags for relative and absolute quantification (iTRAQ) on the proteome level. We found that the DNA Replication and Cell Cycle were the top-2 most significantly downregulated signaling pathways following the DIM+DFMO treatment. Correspondingly and interestingly, these two pathways were the top-2 upregulated ones in clinic ESCC tumors. Moreover, the involved differentially expressed genes (DEGs) including MCM2, MCM3, MCM5, MCM6, MCM7, CDK1, and LIG1 were all inversely downregulated by DIM+DFMO treatment. In the limited clinical study in two ESCC cases, the administration of DIM (250mg) +DFMO (500 mg) once daily showed favorable results, including alleviated swallowing difficulties, decreased blood tumor markers (CA19-9, CA15-3 and AFP), and no severe toxicity in at least one month progression free survival period. We concluded that DIM+DFMO is a promising therapeutic combination for ESCC treatment via the suppression of DNA Replication and Cell Cycle activities. However, these therapeutic effects should be verified in large cohort clinical trials with sufficient cases.

## Introduction

ESCC, the most common pathohistological type of EC, is a serious malignant disease with a high mortality rate; the worldwide five-year survival rate is only 15-20% 1. The conventional chemotherapies for ESCC have limited efficacy and severe side effects, the outcomes of which are also far from satisfactory. Few effective targeted therapies have been established to date, and patients with late-stage ESCC have limited therapeutic options 2. Our previous studies have illustrated that the highly genetic heterogeneity nature of ESCC tumors is also a major obstacle for the development of targeted therapies for patients 3. Through the analysis of the ESCC expression profiles derived from TCGA, we found that DNA Replication and Cell Cycle were the top two most significantly upregulated pathways. Suppression of the dysregulated pathways is one potential effective strategy for screening potent anti-ESCC therapies. Here, a combination of compounds was studied to suppress these dysregulated pathways in ESCC.

Indole compounds have also been found to act against various cancer types including breast, esophageal, gastric, colorectal, liver, and pancreatic cancers 4-8. The compound DIM, as the dimeric product of natural indole-3-carbinol in cruciferous vegetables, has been shown to cause G1 phase cell cycle arrest and induce apoptosis by activating caspase-9 in ESCC cells 9. Recently, *in vivo* and *in vitro* studies on ESCC showed that DIM could reverse epithelial-mesenchymal transition (EMT) process via the modulation of aryl hydrocarbon receptor (AHR), and DIM could inhibit ESCC metastasis through repressing RhoA/ROCK1-mediated COX2/PGE2 pathway 10. Additionally, the AHR modulation by DIM could also inhibit ESCC progression via repressing COX2/PGE2/STAT3 axis 11. We also demonstrated that 3,3'-diindolylmethane (DIM) is a novel COX1/2 and ERK1/2 inhibitor 12.

Eflornithine (DFMO) is an irreversible inhibitor of ornithine decarboxylase (ODC), which is the first rate-limiting enzyme in the polyamine biosynthesis pathway 13. DFMO is an important anti-protozoa drug to treat African sleeping sickness via the inhibition of polyamine metabolism, which is essential for the protozoa survival 14. Increased levels of polyamines are closely associated with numerous cancers 15, 16. DFMO shows considerable anti-proliferative effects in many types of cancer including neuroblastoma, pancreatic cancer, and intestinal carcinoma 17-19. In ESCC, DFMO showed anti-proliferation effects and also induced apoptosis of cell lines and tumors from patient-derived xenograft mouse model, indicating that ODC is a promising target 20. ODC inhibition by DFMO treatment was found to suppress the development of precancerous esophageal lesions in an N-nitrosomethylbenzylamine-induced rat model via downregulating p38a, ERK1/2, and AKT/mTOR/p70S6K signaling pathways, demonstrating the potential chemoprevention effect of DFMO 21. However, the effectiveness of DFMO as a therapeutic reagent has been modest, which was generally found to exert cytostatic effects on mammalian cells and tissue 22. The recent clinical trials of DFMO investigate its chemopreventional efficacy in several epithelial malignancies. In a randomized placebo-controlled trial, DFMO (500mg/day) induced a decrease of prostate putrescine levels and prostate growth rate, with few severe toxicities including ototoxicity 23. In a double-blinded, placebo-controlled trial, DFMO (500 mg/day) plus aspirin (325 mg/day) was associated with significant reduction in the median number of rectal aberrant crypt foci compared with placebo 24. In another randomized placebo-controlled, double-blind trial, a combination of DFMO (500mg/day) and sulindac (150 mg/day) markedly reduced the recurrent adenomatous polyps and rectal mucosal polyamines 25, 26.

DIM in combination with other drugs was much more effective than monotherapy against pancreatic cancer and colon cancer 27, 28. The combination of DFMO and other compounds also effectively inhibited pancreatic cancer cell growth and lung carcinogenesis 29, 30. We screened a variety of bioactive compounds (natural and synthesized) to test the combined efficacy against ESCC. We found that DIM+DFMO combination was much more effective against ESCC than either compound alone. Interestingly, DNA Replication and Cell Cycle as the top two most significantly upregulated pathways in ESCC patients were both inversely downregulated by DIM+DFMO treatment. Favorable efficacy of DIM+DFMO were observed in a limited clinical trial, including alleviated typical ESCC symptoms, decreased blood tumor markers, and no severe toxicity in the at least one month progression free survival period. The optimistic changes promoted the effectiveness and safety of DIM+DFMO treatment for ESCC patients. Further studies are required to certify whether these favorable preclinical and clinical changes could benefit ESCC patients.

## Materials and Methods

### The expression data of ESCC patients derived from TCGA database

The expression data of 81 patients with ESCC and 11 normal tissue samples were downloaded from TCGA database and cited for reference purposes; these were compared with the proteomic results obtained in our current study. Prior to downloading the dataset of ESCC patients from TCGA database, transcript read count files differentiating squamous cell carcinoma from adenocarcinoma were downloaded from the TCGA database (https://portal.gdc.cancer.gov/repository; Primary Site Filter: Esophagus, Diagnoses Morphology Filter: 8070/3, 8071/3, and 8083/3 are classified as Squamous Cell Carcinoma); patient tumor samples (N=81) fitting this criteria were chosen for inclusion. Initial diagnoses of all patients participating in the study were made between 2001-2013; patient samples were processed for sequencing between 2011-2014. Similarly, transcript read counts derived from solid normal esophageal tissue samples (N=11) were identified using the Samples Sample type filter on the TCGA website. To identify the genes differentially expressed between normal and ESCC tissues, read count data was transformed according to the instructions listed within the DESeq2 user manual and processed using the DESeq2 algorithm in the R programming environment.

### Compounds and reagents

DIM compound (CAS: 1968-05-4) was purchased from Sigma (BCBP4381V-D9568, Switzerland), and DIM capsules (DIM-250, 30 vegetarian capsules per bottle/30 servings) were purchased from the Olympian Labs. Inc. (Phoenix, AZ, USA). DFMO (CAS: 68278-23-9) was purchased from Dahua Pharmaceutical Co., Ltd (Wuhan, China).

### *In vivo* PDX models for animal study

CB17/Icr-Prkdc^scid^/IcrlcoCrl mice were purchased from Vital River Laboratory Animal Technology Co., Ltd. (Beijing, China), and were utilized as carriers for the establishment of PDX model. All mice were housed in individually ventilated caging systems (#FSIVC-3318DA, Suzhou Fengshi Laboratory Animal Equipment Co., Ltd) with regular chow and water in a specific pathogen-free environment. The clinical characteristics of the three ESCC cases EG9, EG30, and EG37 were illustrated (Table 1). Fresh ESCC PDX tissue of approximately 200 mm^3^ was implanted into mice that were 4~6 weeks old and weighed 17~19 g; 0.4% pentobarbital sodium (Sinopharm Chemical Reagent Co., Ltd) was used as an anesthetic. Before the tumor implantation into the mice, the H&E staining of the tumor tissue obtained after the mice sacrifice would be examined and compared with the original patient tumor tissue, to certify the viability of the tumor cells and consistency of pathological morphology. After seven days of wound recovery, the mice were randomly divided into four groups: vehicle, DIM-100 mg·kg^-1^ 12, DFMO-2% (W/V) 31, 32, and DIM 100 mg·kg^-1^ + DFMO 2% (W/V) based on tumor volume (tumor volume = length × width^2^ × 0.5). DFMO (2% W/V) was dissolved in the mice's drinking water, while DIM was dissolved in ultrapure water and administrated via oral gavage at 100 mg·kg^-1^. All the groups were treated daily without intermissions. The animals were sacrificed after an average of 63 days, tumor tissues were harvested for hematoxylin and eosin staining and western blotting. The animal study protocol was examined and approved by Human Subjects Institutional Review Board at the China-US (Henan) Hormel Cancer Institute. There were twelve mice for each group in EG37, and ten mice for each group in EG9 and EG30 at the beginning of the animal experiment. However, the final number of tumor tissue presented after sacrifice may be less than the number of the mice. Because some tumor tissue would disappeared gradually after implantation, which potentially attributed to the spatial and inter-sample heterogeneity properties of tumor tissue. Additionally, in PDX mice model, tumor grew from one small piece of tumor tissue and these pieces of tumor tissue come from different tumor carriers (PDX mice). There is the inter-sample heterogeneity across different mice, and the spatial heterogeneity of one tumor tissue. Even we strictly controlled the size, shape, and weight of this small piece tumor tissue before inoculation into the SCID mice, the final size of the tumor still with inevitable variances.

### Western blotting assay

Tumor tissues extracted from the PDX mice that were preserved at -80 °C were homogenized and lysed in RIPA buffer (#R0010, Solarbio) containing 1 mM PMSF (#P8340, Solarbio) at 20 mg of sample per 200uL of the lysis buffer according to the manufacturer's instructions (#R0020, Solarbio). Next, the supernatants were collected by centrifugation at 14,000* g* for 5 min. For cancer cell lines, the medium was discarded and the cells were washed with PBS, after which they were detached after rinsing with RIPA buffer containing 1 mM PMSF. Following the preparation of the tissue or cell lysates, their protein concentrations were quantified using a BCA kit (#PC0200, Solarbio). Next, 30 μg of whole protein lysate was loaded into each lane of an SDS-polyacrylamide gel, and the samples were separated for 50-90 min with 100-150 V. The separated proteins were then transferred to Immobilon®-P polyvinylidene fluoride membranes (#IPVH00010, Merck) using 100 V for 60-120 min at 4 °C. The membranes were then washed and blocked in 5% nonfat milk (#232100, BD) for 2 h, after which they were incubated with primary and secondary antibodies. The primary antibodies included anti-MCM2 (1:1000 dilution, #ab4461, Abcam), anti-MCM7 (1:1000 dilution, #3735, CST), anti-CDK1 (1:1000 dilution, #77055, CST), anti-LIG1 (1:1000 dilution, #177946, Abcam), and anti-GAPDH (1:1000 dilution, #AB-P-R001, Goodhere Biotech). Secondary antibodies included goat anti-mouse IgG-HRP (1:5000 dilution, #sc-2005, Santa Cruz) and goat anti-rabbit IgG-HRP (1:5000 dilution, #sc-2004, Santa Cruz). The protein bands were illuminated using electro enhanced chem-illuminescence (#P0018, Beyotime) using the manufacturer's protocol, and the membranes were finally scanned and analyzed in an Amersham Imager 600.

### LC-MS/MS isobaric tags for relative and absolute quantification (iTraq) assay

The *FG,* iTraq Reagent-8 plex multiplex kit (#4381663, AB) was used for quantitative proteomics according to the manufacturer's instructions. Tissues from two patients (EG30 and EG37) were chosen to perform the iTraq. Three tumor tissues from eight groups (EG30-Con[trol], EG30-DIM, EG30-DFMO, EG30-DIM+DFMO; EG37-Con, EG37-DIM, EG37-DFMO, and EG37-DIM+DFMO) were selected, and 30 mg of tissue was dissected from each tumor. A total of 90 mg from each group was pooled in a single 1.5 mL tube, and samples from each group were then processed for protein extraction and quantified via the BCA kit as described above for western blotting. Next, 200 ug of protein from each group was trypsinized, and 100 ug of peptides with N-termini (-NH2) or side chain amines (-NH-) were made to covalently bond with isobaric labeling tags of varying masses (113, 114, 115, 116, 117, 118, 119, and 121, respectively). The labeled peptide from the eight tubes were pooled and mixed for fractionation via high pH reversed-phase liquid chromatography (#1260 Infinity, Agilent Technologies). Forty-eight fractions were collected and combined into 12 tubes symmetrically, and 8uL from each of these 12 samples was loaded and analyzed via MS/MS (ekspertTM Nano LC 415-AB SCIEX Triple TOF 5600). Fragmentation data from the tandem mass spectrometry spectrum were searched in the ProteinPilot database for identification.

### Bioinformatic data analysis

The DAVID Bioinformatics Resources 6.8 software (NIAID/NIH) from the Laboratory of Human Retrovirology and Immunoinformatics was used for functional annotation and KEGG signal pathway enrichment 33, 34. The KEGG pathway is a collection of manually drawn pathway maps representing current knowledge on molecular interaction, reaction, and relationship networks 35. The gene set enrichment analysis (GSEA) method was used to analyze the expression data of patients with ESCC from TCGA. GSEA is a computational method that determines whether an a priori defined set of genes shows statistically significant, concordant differences between two biological states or phenotypes 36, 37. The open source software platform Cytoscape and the STRING database were used for visualizing complex networks and integrating them with attribute data 38; these were also used for protein-protein association networks with increased coverage to support functional discovery using genome-wide experimental datasets 39. The online Venn software was used to analyze overlaps between different groups (http://bioinformatics.psb.ugent.be/webtools/Venn/).

### A pilot study for assessing the clinic relevant activities and toxicities of DIM+DFMO in ESCC patients

Two cases of ESCC patients were enrolled in this pilot clinic study between November 2020 and January 2021, based on the inclusion criteria 1). Patients were pathologically diagnosed with esophageal squamous cell carcinoma (ESCC); 2). Patients have clinical stage of ESCC in the range of T1aN0M0-T3N0M0; 3). Patients have no basic disease and other types of cancer; 4). Patients have not received neoadjuvant chemoradiotherapy or other therapies before surgery. Informed consent was signed voluntarily by all subjects. The subject characteristics are shown in Table 2. Patients displaying a clinical response were allowed to remain on treatment until disease progression occurred or mutual decision of their physician and their family. A weekly physical exam was performed to monitor the treatment related toxicities. ECOG score was measured before initiation of treatment and after one and two months' treatment. The study proposal and ethics were reviewed and approved by the Human Subjects Institutional Review Board at the China-US (Henan) Hormel Cancer Institute.

### Data and statistical analysis

All bar and line graphs represent means and standard deviations. For comparisons of more than two groups, one-way ANOVA was used. The independent unpaired *t*-test was used to compare differences between two groups. A *P*-value less than 0.05 was considered statistically significant. Data were analyzed using the SPSS (21.0) software (IBM Corp., Armonk, NY). The data and statistical analysis comply with the recommendations on experimental design and analysis in pharmacology 40.

## Results

### Cell Cycle and DNA Replication ranked as the top-2 most significantly dysregulated signaling pathways in ESCC tumor tissues compared with normal tissues

**Figure 1:**
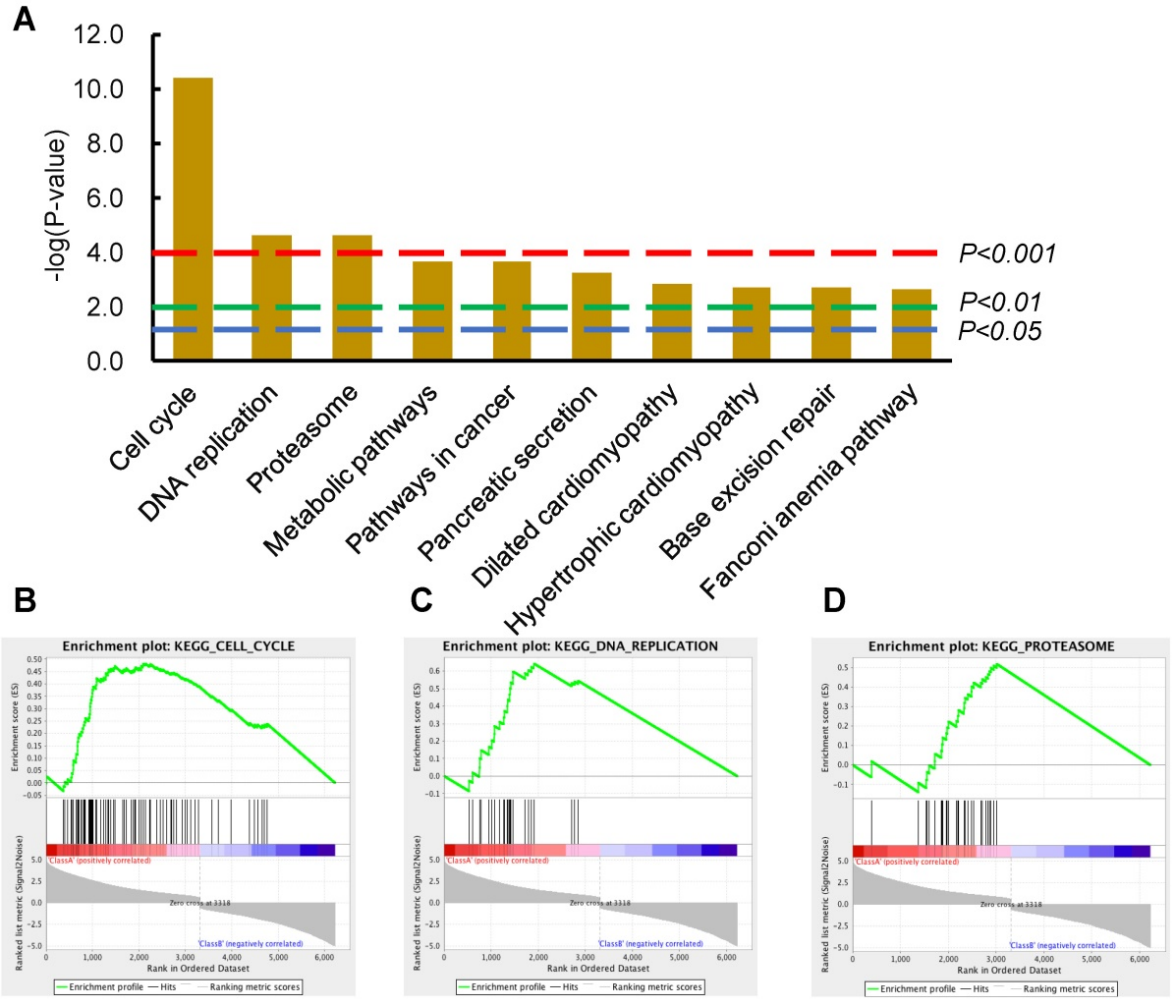
** Cell cycle and DNA replication as the top-2 most significantly dysregulated signaling pathways provided potential therapeutic significance for ESCC patients. (A)** The top-10 most significantly changed KEGG pathways were illustrated in the column graph with -log(*p*-value) as the *Y*-axis. Pathways of columns above (higher than) the dashed red, green, and blue lines indicate *p*<0.001, *p*<0.01, and *p*<0.05 respectively. **(B-D)** The GSEA results of the first three KEGG pathways; the enrichment scores are 0.48, 0.64, and 0.52, the normalized enrichment scores are 2.77, 2.66, and 2.27, the nominal *p*-values are 0.0, 0.0, and 0.0, FDR *q*-values are 0.0, 0.0, and 0.0, and the familywise error rate *p*-values are 0.0, 0.0, and 0.0, in the gene set of Cell Cycle, DNA Replication, and Proteosome, respectively.

An expression profile of DEGs (*P*-value <0.001, fold change >1.2) were derived from comparing 81 ESCC tumor tissues with 11 normal tissues from the TCGA database (details in materials and methods). The top two significantly upregulated signaling pathways were Cell Cycle and DNA Replication from the list of enriched KEGG signaling pathways using DAVID (Figure 1A). To test the reliability of these data, GSEA was also performed to evaluate the significance of these DEGs; again, the dysregulated pathways modulating Cell Cycle, DNA Replication, and Proteasome were enriched from the corresponding DEGs (Figure 1B-D). It is inspired that suppression of these upregulated cellular activities of DNA Replication and Cell Cycle could potentially fight against ESCC and benefit patients. We next sought to verify these findings in ESCC PDX model, to investigate whether suppression of these upregulated Cell Cycle and DNA Replication pathways could inhibit ESCC tumor growth.

### DIM+DFMO combination produced a more potent inhibition effect than single treatment alone in ESCC PDX model

**Figure 2:**
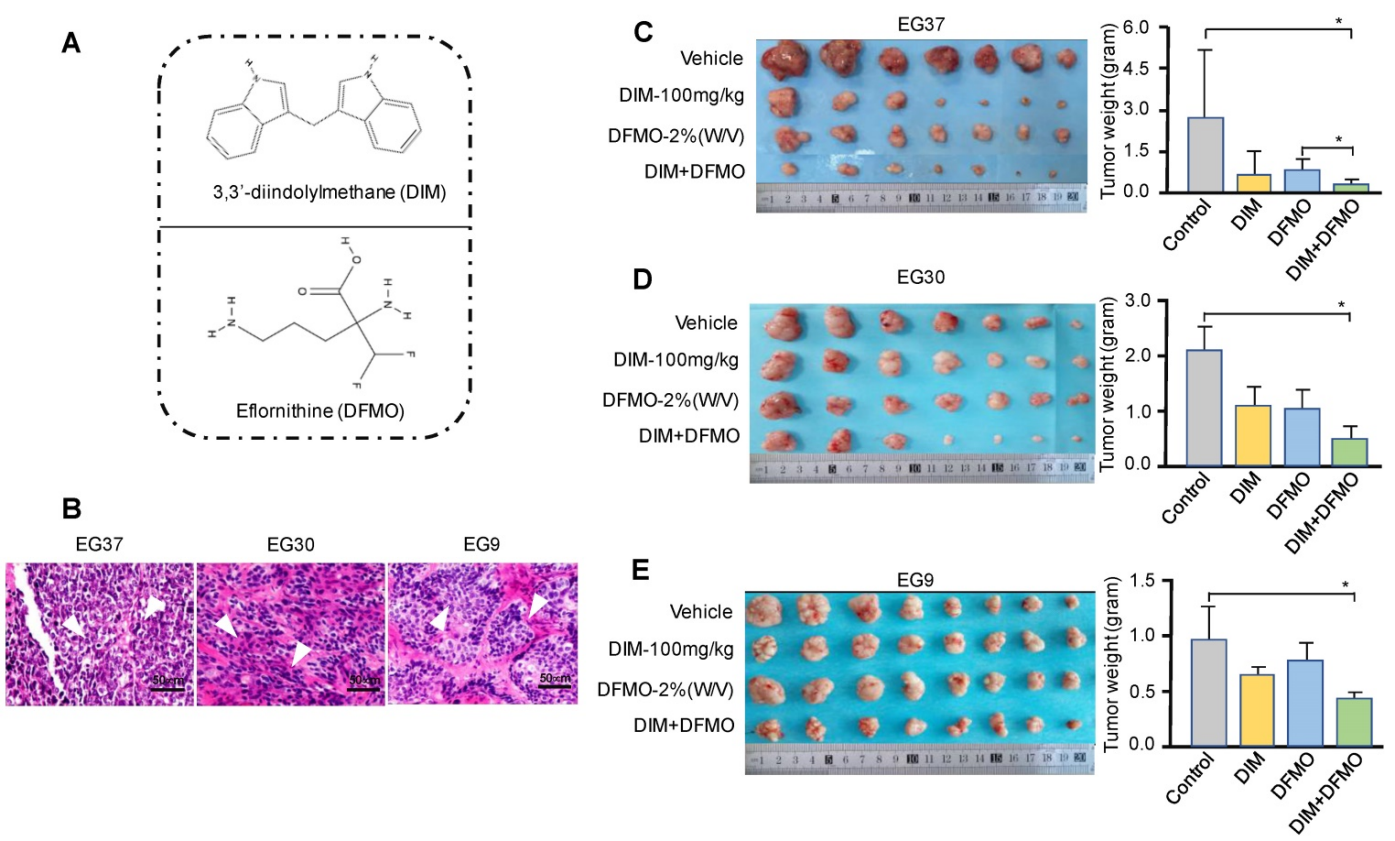
** Administration of combination DIM+DFMO in an ESCC-PDX model showed significant anticancer effects. (A)** The chemical structures of DIM and DFMO were shown;** (B)** HE staining of the three ESCC PDX cases at 200X magnification showed differed differentiation status, scale bar is 50 µm. The white arrows indicated the tumor lesions. **(C)**,** (D)**, and **(E)** showed the *in vivo* results for EG37, EG30, and EG9 ESCC PDX models, respectively. Left images showed representative PDX tumor tissues in the vehicle and treatment groups [DIM-100 mg·kg^-1^, DFMO-2% (W/V), and DIM-100 mg·kg^-1^+DFMO-2% (W/V)] after sacrifice, original complete tumor images could be referred in the Supplemental Figure 1. The right bar graphs show average tumor weights in each group for the three patient PDXs (EG37, EG30, and EG9) after treatment. *0.01<*P*≤0.05, **0.001<*P*≤0.01, *** for *P*≤0.001.

PDXs represent a novel and important tool for preclinical modeling in the study of cancer therapeutics 41, 42. A variety of natural compounds and synthetic compounds were screened in the ESCC PDX model. We found that the combination administration of DIM+DFMO (Figure 2A) possess synergistic and potent anti-ESCC effect *in vivo*. To further verify this discovery, three PDX cases with varied differentiation status (EG37, EG30, and EG9) (Table 1, Figure 2B) were passaged to verify the anticancer effect of DIM (100 mg·kg^-1^), DFMO (2% W/V), and both combined. The animal experiment would be terminated before the largest diameter of any tumor reaches 20 mm. After sacrifice, tumor tissues from the DIM+DFMO groups were obviously smaller than these from the vehicle groups in all the three cases (Figure 2C-E). Moreover, the tumor weights in the DIM+DFMO groups of all the three cases were significantly lower than those in the vehicle groups, and even significantly lower than the single treatment group (DFMO) in the case of EG37 (Figure 2C). The less inhibitory effect of DIM alone is improved after combining with DFMO, which demonstrated the synergistic and inhibitory effect of DIM+DFMO combination on ESCC proliferation. However, the body weights of mice in the four groups were not significantly decreased (data not shown). The above results indicated that the combination of DIM+DFMO could effectively inhibit the ESCC proliferation in the PDX mouse model and was safe *in vivo*.

### A subset of 151 differentially expressed genes commonly shared by two ESCC PDXs were obtained from the relative quantitative proteomic analysis of PDX tumor tissues

**Figure 3:**
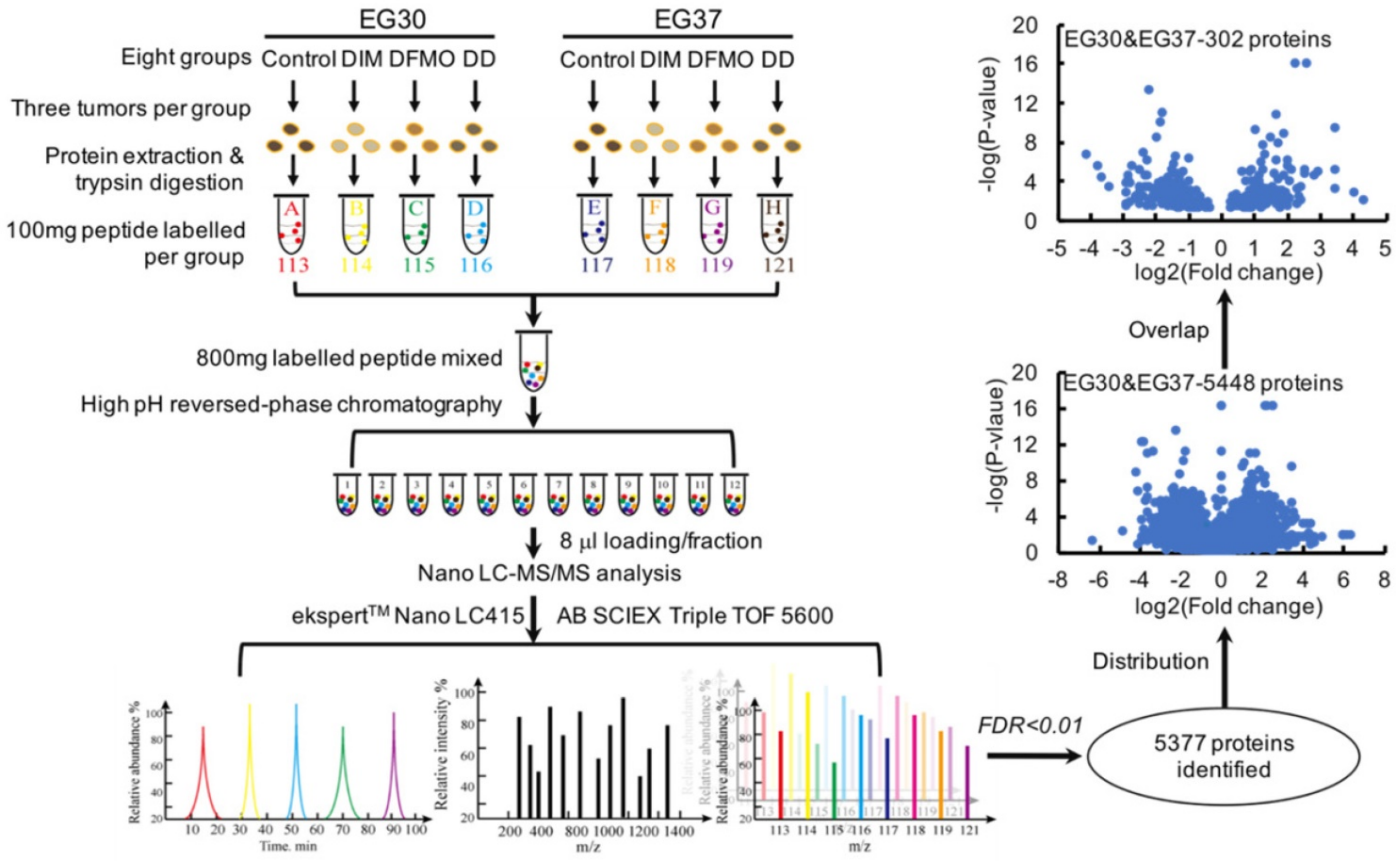
** The experimental workflow and data processing for relative proteomic quantification of the PDX tumor tissues were quality controlled.** The workflow for relative proteomic quantification of the eight groups of tumor tissues from the two PDX cases. 5377 individual proteins were identified at the cut-off of *FDR*<0.01. Moreover, 5488 proteins were obtained by summing the same shared protein numbers from the six datasets of treatment group/vehicle group for both cases. With a fold change>1.2 and* p*<0.05 as the screening thresholds, a subset of 151 individual DEGs was produced via overlapping the subset of proteins from the same treatment group/vehicle group of EG30 and EG37, these 151 DEGs were commonly shared by the two cases, meaning 302 (302 = 151*2) proteins were obtained for the two cases.

The combinational use of DIM+DFMO showed potent anti-ESCC effects *in vivo*. To investigate the underlying mechanisms of this combined effect, LC-MS/MS was used to study the relative quantitative proteomes of PDX tumor tissues from the vehicle groups and three treatment groups in EG30 and EG37 (Figure 3). In total, 5,377 individual proteins (False Discovery Rate [FDR] <0.01) were identified. Three datasets were produced from comparing treatment groups to vehicle group in each of the two PDX cases, and the six datasets for the two cases were EG30-DIM/Con, EG30-DFMO/Con, EG30-(DIM+DFMO)/Con, EG37-DIM/Con, EG37-DFMO/Con, and EG37-(DIM+DFMO)/Con. There were 850 differentially expressed genes (DEGs) shared by the three EG30 datasets and 966 by the three EG37 datasets. Altogether, 5,488 (5,488 = 850 * 3 + 966 * 3) individual DEGs were plotted in a volcano map by using the two parameters of each DEG (log_2_[Fold change] and -log_10_[P-value]). To increase the reliability and credibility of the proteomic study, DEGs in biologically replicated datasets from two PDX cases were filtered and compared for overlap. Finally, a subset of 151 (151 * 2 = 302) DEGs commonly shared by the two PDX cases were obtained using the parameter of log_2_(Fold change) and -log_10_[P-value]. These 151 DEGs reflected the anti-ESCC effects of DIM/Con, DFMO/Con and DIM+DFMO/Con on the proteomic level.

### DNA Replication and Cell Cycle signaling pathways were significantly downregulated following the combined administration of DIM+DFMO in ESCC PDX model

**Figure 4:**
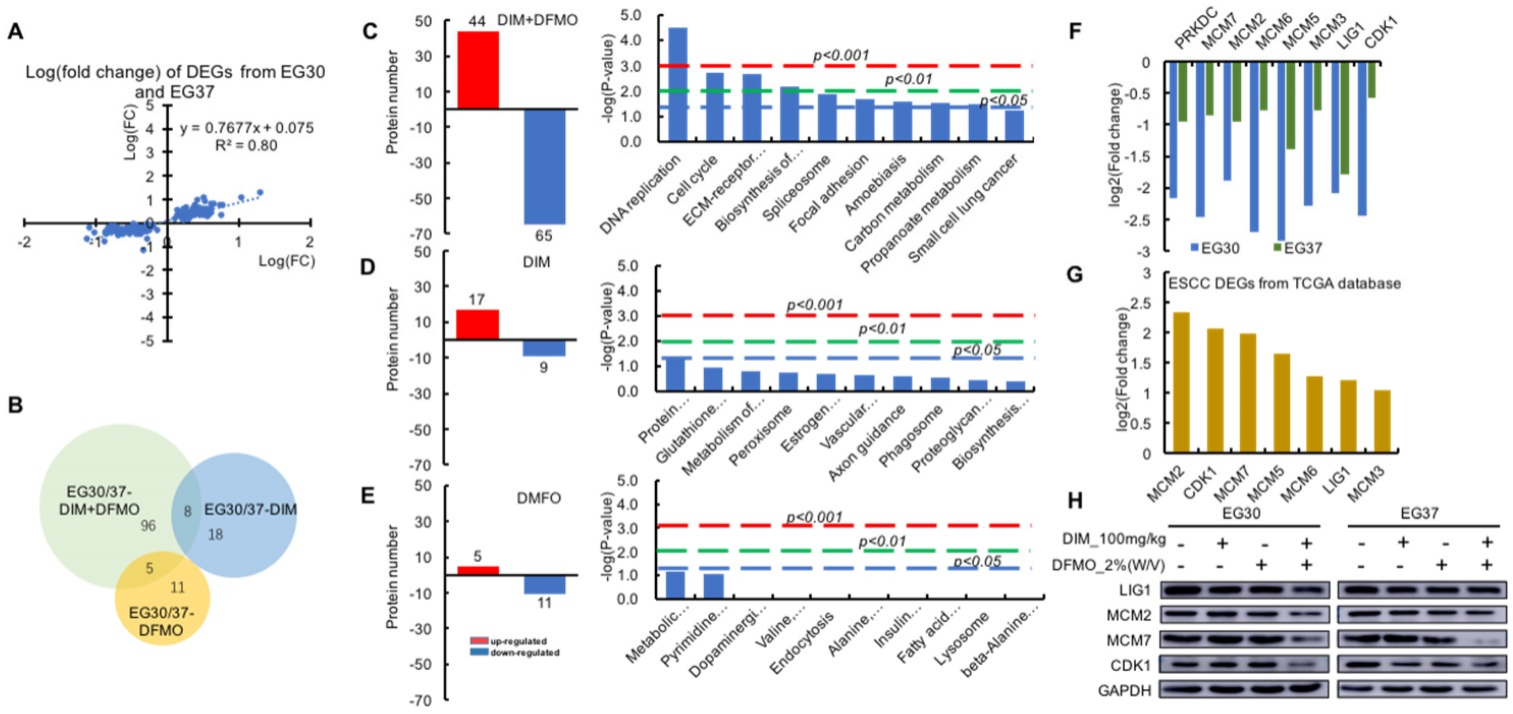
** The DNA replication and cell cycle activities were significantly downregulated by the combined administration of DIM+DFMO, which were highly upregulated in ESCC patients. (A)** The positively and linearly correlated expression pattern of the 151 DEGs from the two cases of ESCC PDX. **(B)** A proportionally scaled Venn diagram were created via overlapping the three datasets, which were 109 DEGs in the EG30/37-DIM+DFMO group, 26 DEGs in the EG30/37-DIM group, and 16 DEGs in the EG30/37-DFMO group. Eight proteins were commonly shared by EG30/37-DIM+DFMO and EG30/37-DIM; five were shared by EG30/37-DIM+DFMO and EG30/37-DFMO. **(C-E)** The left graphs showed the numbers of upregulated proteins and downregulated proteins in the DIM+DFMO group (109 proteins), DIM group (26 proteins) and DFMO (16 proteins), respectively. The right column graphs illustrated the top-10 pathways enriched from the corresponding proteins in each group with -log(*p*-value) as the parameter, respectively. Pathways of columns above (higher than) the dashed red line, dashed green line, and dashed blue line refer to *p*<0.001, *p*<0.01, and *p*<0.05 respectively, indicating the signaling pathways with different degree of significance. **(F)** The relative expression levels (log_2_[fold change]) of all the eight significantly downregulated proteins in the enriched DNA Replication and Cell Cycle pathways were shown from the EG30-DIM+DFMO/Con and EG37-DIM+DFMO/Con group. **(G)** Seven out of the eight downregulated proteins in (F) were significantly upregulated (-log_2_[fold change]) from the first two KEGG pathways in Figure 1A. **(H)** The expression levels of LIG1, MCM2, MCM7, CDK1, and GAPDH were presented from different groups of two ESCC PDX cases, respectively.

To test the reproducibility of the therapeutic effects and stability on the two biologically replicated PDX cases, two sets of 151 DEGs were plotted and showed a highly positive and linear correlation (*R^2^* = 0.8) on the proteomic level from the two cases (Figure 4A). Out of the 151 DEGs, a proportionally scaled Venn diagram was produced to demonstrate the common and unique proteins between different datasets (Figure 4B). 109 DEGs were contained in the DIM+DFMO/Con group, whereas only 26 and 16 DEGs were included in the DIM and DFMO single treatment groups, respectively, which illustrated more DEGs were produced by the combinational treatment of DIM+DFMO than single compound. Furthermore, eight and five proteins were shared by the datasets of DIM+DFMO/Con and DIM/Con, DIM+DFMO/Con and DFMO/Con, respectively.

To determine the mechanisms involved in the anti-cancer effect of DIM+DFMO, KEGG pathways were enriched from the corresponding DEGs (Figure 4C-E). Nine signaling pathways were significantly (*P* < 0.05) influenced by DIM+DFMO treatment; the top two were DNA Replication and Cell Cycle regulation (*P* = 0.00003 and 0.002, respectively). In contrast, none of the pathways in the DIM-only or DFMO-only group were statistically significant, which indicated that a single compound alone was not as effective as the combined treatment.

In total, eight individual proteins (PRKDC, MCM2, MCM3, MCM5, MCM6, MCM7, LIG1, and CDK1) were presented in the DNA Replication and Cell Cycle pathways after the DIM+DFMO treatment (Figure 4C). These eight proteins were all downregulated after treatment with combination DIM+DFMO (Figure 4F), and these downregulation were verified via western blotting (Figure 4H). The top-2 most upregulated proteins (MCM2 and CDK1) were also verified in two ESCC cell lines (Kyse30 and Kyse70) (Supplemental Figure 2). However, for the heterogeneity of gene expression across different samples and cancer cell lines, the effect of DIM+DFMO on the gene expression is also with little variances. In clinical samples, seven (MCM2, CDK1, MCM7, MCM5, MCM6, LIG1, and MCM3) of these eight proteins were found to be significantly overexpressed in ESCC patients, with MCM2 and CDK1 as the top-2 most upregulated proteins (Figure 4G). The aforementioned findings suggested the significance of downregulating these dysregulated proteins for a therapeutic purpose in ESCC patients.

Since DNA Replication, Cell Cycle pathways, together with the corresponding eight proteins (Figure 4G) were all significantly downregulated following the combined administration of DIM+DFMO in the two replicate ESCC PDX cases (Figure 4C). These anti-cancer effects were accurately corroborated by reversing the upregulation of DNA Replication and Cell Cycle signaling pathways occurred in ESCC patients (Figure 1). These data pointed to a potential synergistic anticancer effect of combination DIM+DFMO against ESCC through the inhibition of cell cycle and DNA replication pathways.

### Optimistic results from the two ESCC cases indicated promising therapeutic effects of the DIM+DFMO for ESCC treatment, which should be thoroughly investigated in large cohorts of clinical trial

**Figure 5:**
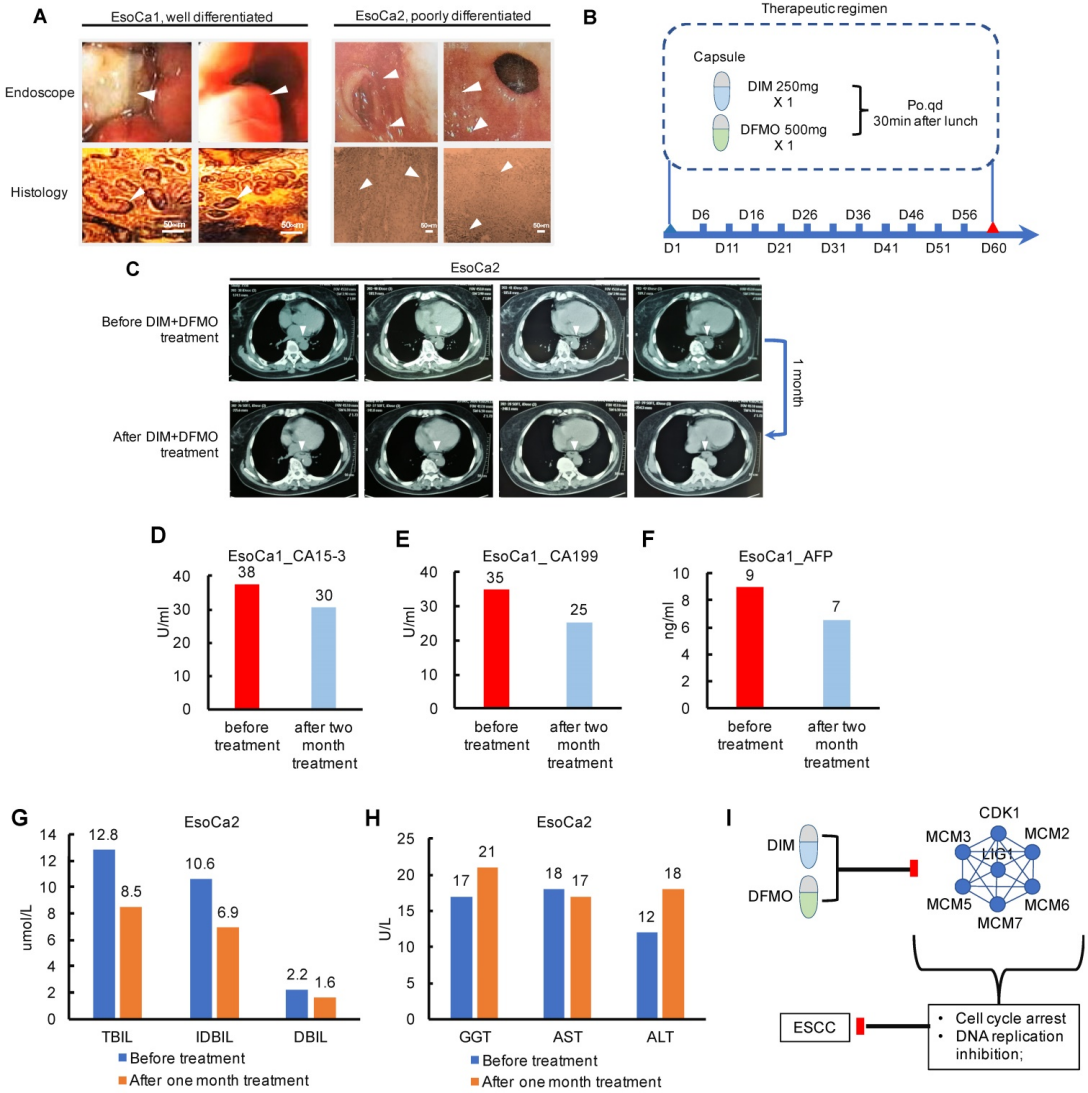
** Pioneering investigation of the anti-cancer effect of DIM+DFMO in two cases of ESCC patients. (A)** Representative endoscopic images and histological images (H&E staining) of sampled biopsies were illustrated from the two ESCC cases, respectively. White arrows indicated the tumor lesions. Scale bar is 50 µm. **(B)** The therapeutic schedule of DIM+DFMO applied for the two ESCC patients; **(C)** Representative CT images taken before DIM+DFMO treatment and after one month treatment, white arrows indicate the tumor lesions in the ESCC patient; **(D-F)** The measurement of blood tumor markers from the ESCC patient before DIM+DFMO treatment and after two month treatment; **(G-H)** The common liver function tests from the ESCC patient were presented before treatment and after one month DIM&DFMO treatment; **(I)** The hypothesis of anti-cancer mechanisms of DIM+DFMO for ESCC patients; Normal range of CA15-3 and CA199 is 0-35 U/ml; AFP (alpha-fetoprotein) is 0-10 ng/ml; TBIL (total bilirubin), 3.42-20.5 umol/L; IDBIL (indirect bilirubin), 2-17 umol/L; DBIL (direct bilirubin), 0-6.84 umol/L; GGT (gamma-glutamyl transpeptidase), 7-50 U/L; AST (aspartate transaminase), 8-40 U/L; ALT (alanine transaminse), 0-40 U/L.

Since the combination of DIM+DFMO was demonstrated being able to suppress ESCC proliferation via downregulating DNA Replication and Cell Cycle activities, which was also corroborated by corresponding dysregulated pathways from TCGA ESCC patients. It is essentially necessary to translate these favorable pre-clinical findings to clinic and benefit ESCC patients. Two ESCC cases were enrolled in a limited clinical study to test the dose tolerance, side effect and potential toxicity of the DIM (250 mg/day) plus DFMO (500 mg/day) treatment (Table 2). The dosage of DIM 43-45 and DFMO 23-25 based on the dosages reported in literatures and clinical trials. EsoCa1 and EsoCa2 were both in the clinical stage of T2N0M0. EsoCa1 and EsoCa2 were identified as well differentiated (G1) and poorly differentiated (G3), respectively (Figure 5A). The same therapeutic schedule was performed for both cases (Figure 5B). At the time point of one month and two month, CT scanning, blood tumor marker test, liver and kidney function test were carried out with representative results shown (Figure 5C-H).

Both the two patients claimed that the swallowing difficulties and sore were obviously alleviated after the first three days' treatment. The fatigue state had been greatly improved and outdoor exercises had increased gradually. The food intake had changed gradually from liquid to semisolid, and the vomiting caused by swallowing difficulties had largely improved after one week treatment. However, these fast improvements, orally reported by these two patients, may be partially attributed to the placebo effects or psychological effects. Moreover, the progression free survival lasted at least for one month verified by chest CT examination (Figure 5C). These potential placebo effects would be considered and eliminated by the double blind plus positive control (Taxol+Platin) in the future design of large cohort clinical trial. The anti-ESCC effect from DIM+DFMO treatment should have been maintained until the ending point of the therapeutic schedule, validated by the decreased blood tumor markers compared with that at the diagnosis (Figure 5D-F). Additionally, no severe side effect or severe toxicity were observed during the whole therapeutic schedule for both cases, including the liver function test (Figure 5G-H). The values of total bilirubin, indirect bilirubin, and direct bilirubin representing the liver functions all decreased (Figure 5G), and the values of these three indicators were all within the normal ranges before and after one month's treatment, indicating no obvious influence of DIM+DFMO on the liver function of the ESCC patient. The potential anti-ESCC mechanism following DIM+DFMO treatment was that through downregulation of the Cell Cycle and DNA Replication associated proteins (MCM2/3/5/6/7, CDK1 and LIG1), the hyperactive activities of these signaling pathways were suppressed and lead to the inhibition of tumor proliferation in ESCC patients (Figure 5I).

## Discussion

### Current status of diagnostic and therapeutic of esophageal squamous cell carcinoma

In 2020 Globocan estimates, 604,000 new cases and 544,000 deaths of esophageal cancer occurred in 2020, which ranks seventh and sixth in terms of incidence and mortality worldwide, respectively. Geologically, Eastern Asia exhibits the highest regional incidence prevalence for both women and men, partially for the largest burden in China, followed by Southern Africa, Eastern Africa, Northern Europe, and South Central Asia 46. ESCC is the most common histological type of EC world-wide, with higher incidences in developing countries 1. In the pT1a/b-m3 ESCC patients, the 5-year overall survival rates were 90-100% for endoscopic resection-esophagectomy and 75-85% for endoscopic resection-chemoradiotherapy 47. However, ESCC is usually diagnosed in advanced, metastatic stages, and its therapeutic outcomes are relatively poor with five-year overall survival rates of approximately 10% and five-year post-esophagectomy survival rates of 15-40% 48. The neoadjuvant chemotherapy (nCT) or chemoradiotherapy (nCRT) as a novel therapy before surgery has been investigated in ESCC patients. In a phage III multicenter, randomized, open-label clinical trial, the nCRT plus surgery achieved a 43.2% pathologic complete response rate, with a better median overall survival and prolonged disease-free survival compared with surgery alone among patients with locally advanced ESCC, with acceptable and manageable adverse events 49. Further, a prospective, multicenter, open-label, randomized clinical trial compared the safety and efficacy of nCRT with neoadjuvant chemotherapy (nCT) in 264 patients with locally advanced ESCC (cT3 to T4aN0 to 1M0), the ESCC patients received nCRT showed a higher pathologic complete response rate than those patients received nCT, and with a higher rate of negative lymph node metastasis rates 50.

Immunotherapy as an optional neoadjuvant therapy has also been studied in ESCC patients. In a phase II clinic trial, tislelizumab (PD-1 antibody, 200mg i.v. Q3W) plus chemotherapy (cisplatin + fluorouracil) was utilized as the first-line treatment for advanced ESCC. The observed objective response rate and disease control rate were 46.7% and 80%, respectively, demonstrating durable responses with manageable tolerability (anemia, decreased appetite, nausea, and asthenia) 51. Consistently, in a single-arm phase 2 clinical trial (ESONICT-1), 30 patients received 2 cycles of neoadjuvant immunochemotherapy (nICT, cisplatin+paclitaxel+sintilimab). The pCR rate was 21.7%, and MPR rate was 52.2% 52. Though the immunotherapy was associated with a significant improvement in overall survival compared with chemotherapy in ESCC patients 53, the toxicities, primary immune escape and secondary immune escape following immunotherapy make it far from “the cure” 54. The limited improvement of treatment outcomes indicated that alternative strategy should be investigated to effectively combat ESCC. Through analyzing the expression profile of ESCC in clinic, we have found that DNA Replication and Cell Cycle are the top-2 most significantly upregulated pathways. Therefore, natural and synthesized compounds could significantly suppress the corresponding dysregulated pathways have been screened in our study, for the purpose of identifying effective anti-ESCC reagents.

### DIM+DFMO combination could potently suppress dysregulated DNA Replication and Cell Cycle activities

The proteomic results provided comprehensive information regarding the mechanisms underlying the anticancer effects of DIM+DFMO treatment. A variety of pathways were significantly changed only in the DIM+DFMO group rather than the single treatment groups. Moreover, the signaling pathways that were altered by DIM+DFMO were not necessarily a combination of the pathways influenced by each compound alone. Interestingly, the DNA Replication and Cell Cycle were the top-2 most significantly downregulated pathways only evidenced in DIM+DFMO group. The DEGs involved in these pathways were all downregulated. Moreover, majority of all these DEGs were found to be significantly upregulated in clinical ESCC tumors. Above results indicated that the downregulation of DNA Replication and Cell Cycle pathways underlies the anti-proliferation mechanism of this combined approach, which was also corroborated by the reversing the upregulation of these pathways in ESCC patients.

Of the Cell Cycle DEGs revealed in the proteomic results, CDK1 and MCM2/3/5/6/7 were significantly associated with ESCC carcinogenesis. CDK1 is a potential EC marker and a putative therapeutic target 55. Moreover, CDK1 downregulation was related to ESCC radio-sensitivity 56. MCM2 was proved as a reliable marker for assessing tumor growth, aggressiveness, and prognosis in ESCC patients 57. Furthermore, these DEGs derived from clinical ESCC samples were also significantly enriched in dysregulated DNA Replication and Cell Cycle pathways in ovarian cancer, pancreatic ductal adenocarcinoma, and hepatocellular carcinoma tissues 58-60. Above evidences indicated that these cell cycle- and DNA replication-associated DEGs were closely associated with many types of cancer. Therefore, downregulation of these overexpressed DEGs via DIM+DFMO treatment could be a potential therapeutic strategy for ESCC patients. It is suggested that the combined administration of DIM+DFMO inhibits ESCC growth via a potentially synergistic manner via the suppression of cell cycle- and DNA replication-related signaling pathways.

### The application of DIM+DFMO for ESCC treatment

The biosafety of the currently used dosages of DIM+DFMO were thoroughly studied and evaluated in previous clinical trials, which appeared to be rather safe. For instance, varied doses of DIM have been administered in different clinical trials, majorly in the range of 75-300 mg daily, such as 300 mg DIM/day X 14 days for the thyroid proliferative disease, no toxicities observed 43, 108 mg DIM/day X 30 days for postmenopausal women with a history of early-stage breast cancer, and no significant side effects were reported 61, 150 mg DIM twice daily X 12 months for breast cancer patients prescribed tamoxifen, and minimal adverse events were reported 45. Generally, DFMO as a single agent has demonstrated chemopreventive and anti-cancer activities. Moreover, DFMO at less than 0.5 g/m^2^/day (equal to 875 mg/day for 65 kg people) for long-term administration is relatively safe, no systematic toxicities have been evidenced 62. Consistently, no measurable or noticeable toxicity of DIM (250 mg) + DFMO (500 mg) once daily were monitored by hematology and liver toxicity test in the current study.

Combination treatments, especially for chemotherapy, are used against multiple targets to provide synergistic efficacy against malignancy. The combination of gemcitabine/erlotinib/DIM could more effectively inhibit pancreatic cancer cell lines that highly express EGFR, NF-κB, and COX2 than any one of the three agents both *in vivo* and *in vitro* 63. Moreover, a phase III clinical chemoprevention trial revealed that a combination of low oral doses of DFMO and sulindac markedly reduced recurrent adenomatous polyps with few adverse effects when compared to the placebo-treated group 25.

In the current limited clinical trial, two ESCC cases were enrolled to test the anti-ESCC efficacy of DIM+DFMO combination. Typical ESCC symptoms such as dysphagia, oesophageal pain, nausea and vomiting, fatigue, appetite loss, and gradually weight loss, result in significantly decreased life quality of the patients 64. These symptoms were immediately alleviated for both recruited cases within the first week of DIM+DFMO treatment. CA199 is an independent prognostic factor of poor overall survival which may be useful in predicting the therapeutic outcomes for ESCC 65. The serum level of CA199 was decreased from 35 U/mL to 25 U/mL after two month treatment, which indicated the potential efficacy of DIM+DFMO in prolonging the survival time of late-stage ESCC patients. However, these fast improvements, orally reported by these two patients, may be partially attributed to the placebo effects or psychological effects. These potential placebo effects would be considered and eliminated by the double blind plus positive control (Taxol+Platin) in the future design of large cohort clinical trial. It is concluded that DIM+DFMO combination is a potentially promising chemotherapy for ESCC treatment via the suppression of DNA Replication and Cell Cycle activities, which should be validated in large clinical trials before clinical recommendation.

## Supplementary Material

Supplementary figures.

## Figures and Tables

**Table 1:** Demographic and clinical characteristics of three cases of ESCC-PDX

	EG9	EG30	EG37
Sex	Male	Male	Male
Age (years)	66	73	69
Diagnosis	ESCC	ESCC	ESCC
Clinical TNM stage	T2N1M0	T3N0M0	T3N0M0
Histologic grade	G1	G2	G3

**Abbreviations:** ESCC, Esophageal Squamous Cell Carcinoma; Histologic grade: G1, well differentiated; G2, moderately differentiated; G3, poorly differentiated; NA, not available.

**Table 2:** Demographic and clinical characteristics of two ESCC cases

	EsoCa1	EsoCa2
Sex	Male	Female
Age (years)	64	70
Clinic symptom	Swallowing Difficulties/Sore	Swallowing Difficulties/Sore
Endoscope	27~32cm neoplasm	30~35cm neoplasm
Diagnosis	ESCC	ESCC
ECOG status	2	2
Clinical TNM stage	T2N0M0	T2N0M0
Histologic grade	G1	G3
Prior surgery	No	No
Prior chemotherapy	No	No

**Abbreviations:** ESCC, Esophageal Squamous Cell Carcinoma; ECOG, Eastern Cooperative Oncology Group; EGOG 1, symptomatic but completely ambulatory. Restricted in physically strenuous activity but ambulatory and able to carry out work of a light or sedentary nature; ECOG 2, symptomatic, <50% in bed during the day. Ambulatory and capable of all self-care but unable to carry out any work activities. Up and about more than 50% of waking hours; ECOG3, symptomatic, >50% in bed, but not bedbound. Capable of only limited self-care, confined to bed or chair 50% or more of waking hours. G1, well differentiated; G2, moderately differentiated; G3, poorly differentiated; NA, not available.
