# Supplementary material for: 3,3'-Diindolylmethane plus Eflornithine suppress DNA Replication and Cell Cycle in Esophageal Squamous Cell Carcinoma in vivo
Source: J Cancer. 2022 May 16;13(8):2607–19. doi: 10.7150/jca.65506 (PMC9174871; doi:10.7150/jca.65506)
Supplement: Supplementary file 1 — Supplementary figures. [file jcav13p2607s1.pdf]

Supplemental Figure 1~2.

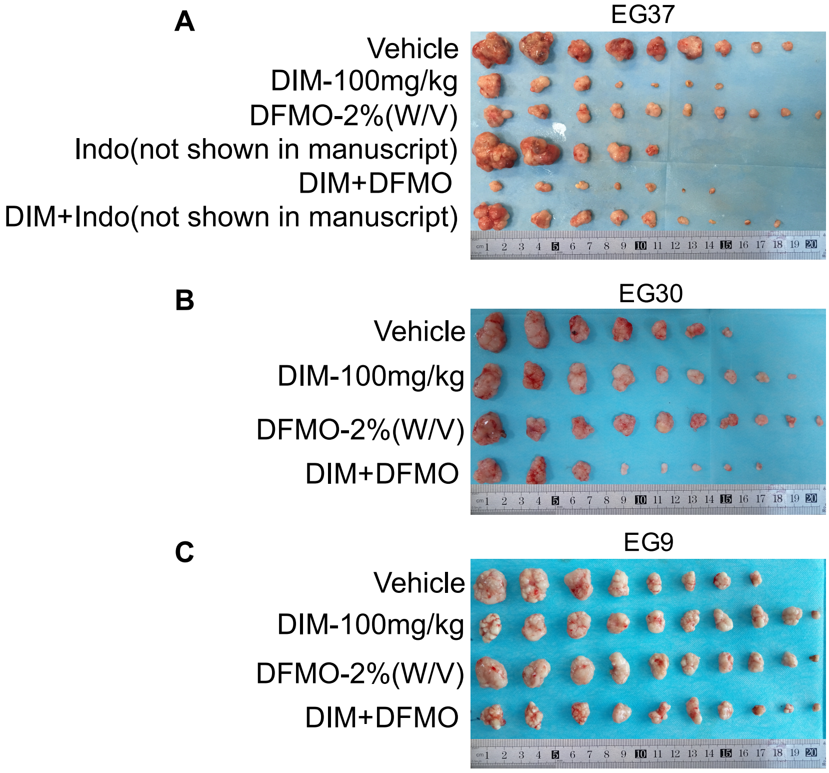

Supplemental Figure 1. (A), **(B)**, and **(C)** showed the results of original complete tumor images for EG37, EG30, and EG9 ESCC PDX models, respectively. The treatment groups in each PDX case were DIM_100mg/kg, DFMO_2% (W/V), and DIM_100mg/kg+DFMO_2%(W/V), respectively.

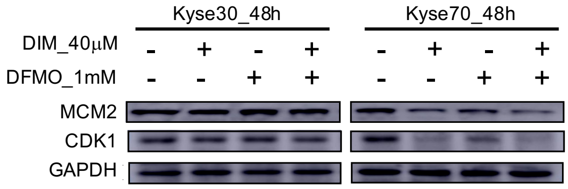

Supplemental Figure 2. The protein expression levels of MCM2 and CDK1 in ESCC cell lines (Kyse30 and Kyse70) were presented after 48h treatment by Vehicle, DIM_40uM, DFMO_1mM, and DIM_40uM +DFMO_1mM, respectively.
